# Attitudes toward dementia and cognitive aging among Syrian refugees resettled in Jordan: a qualitative study

**DOI:** 10.1186/s12889-023-17183-5

**Published:** 2023-11-22

**Authors:** Lana Bridi, Dahlia A. Kaki, Rawnaq Behnam, Xara Khan, Behnan Albahsahli, Nissma Bencheikh, Raghad Aljenabi, Nargis Ahmadi, Rana Dajani, Tala Al-Rousan

**Affiliations:** 1 School of Medicine, University of California, San Diego, San Diego, CA USA; 2 Herbert Wertheim School of Public Health and Human Longevity Science, University of California, San Diego, San Diego, CA USA; 3 School of Medicine, University of California, San Francisco, San Francisco, CA USA; 4 School of Social Sciences, University of California, San Diego, San Diego, CA USA; 5 Department of Biology and Biotechnology, The Hashemite University, Zarqa, Jordan

**Keywords:** LMICs, Prevention, Middle East, Alzheimer’s, Socioecological model, Healthcare, Displacement

## Abstract

**Background:**

Mounting evidence is revealing disparities in cognitive function and heightened dementia risk among refugees, yet research in this area remains scant. Despite bearing most of the world’s refugee burden, limited-resource countries like Jordan are facing challenges when dealing with refugee health. There is a lack of research on the attitudes toward dementia and the cognitive healthcare gaps among refugees in Jordan.

**Methods:**

32 older (≥ 55 years) Syrian refugees resettled in Jordan were recruited through a local community-based organization and interviewed in four focus groups (2 female and 2 male groups). Interviews were transcribed and translated, then coded using inductive thematic analysis.

**Results:**

Mean age of the sample was 60.1 years and 53.1% were female. Only 34.4% rated their memory as good or excellent. Themes were organized using the socioecological model: 1) At the individual level, participants believed high levels of stress, including low socioeconomic status, poor health, and traumatic history from their refugee experience increased their dementia risk. 2) Interpersonally, there is a fear of dementia due to the possible impact and burden on loved ones, particularly with the stigma surrounding dementia. 3) At the community level, participants noted that resettlement in Jordan – with a shared language, religion, and culture – offered protective effects due to facilitated access to social connection, information, and mental health self-care. 4) At the institution and policy level, participants believed older refugees faced restrictive policies for economic aid, healthcare, and employment, presenting a significant barrier to healthy aging.

**Conclusions:**

Findings from this study are the first to examine the attitudes of Syrian refugees in Jordan toward dementia and cognitive aging. These results could provide essential data inclusive of refugees as Jordan develops its National Dementia Plan. Investing in dementia awareness interventions and age-friendly neighborhoods may benefit aging refugees in limited-resources settings.

**Supplementary Information:**

The online version contains supplementary material available at 10.1186/s12889-023-17183-5.

## Background

Rates of forced displacement continue to rise, reaching a staggering 89.3 million by the end of 2021 [1]. Low- and middle-income countries (LMICs) host 83% of the world’s refugees and face many challenges in supporting their refugees’ needs [1–3]. Despite increasing attention to refugee health research in LMICs, the needs of aging/older refugees are understudied [4]. As global health experts call for increased research into supporting the needs of older people in LMICs [5], it is imperative to focus on aging refugee health needs in these countries.

The Syrian refugee population remains the largest globally displaced with 6.8 million refugees [1]. Jordan hosts the second-highest share of refugees per capita globally with 1.36 million Syrian refugees [6]. The influx of older Syrian refugees along with the aging Jordanian and refugee populations are shifting the national disease burdens toward non-communicable diseases and multiple comorbidities [7, 8], with dementia – a category of several progressive diseases such as Alzheimer’s disease affecting memory, cognitive function, and behavior – constituting a growing burden [9, 10]. Moreover, the Jordanian government has explicitly identified care for older refugees to be a “significant challenge” [6]. Dementia is a global health crisis for the aging population with 50 million people affected globally, the majority of whom reside in LMICs. Many LMICs are struggling to develop National Dementia Plans to address this growing burden [10, 11]. The Middle East and North Africa (MENA) regions, with close to 3 million cases of dementia in 2019, are predicted to experience the largest increase in dementia cases in the world at 367% due to population aging, leaving countries such as Jordan most vulnerable to challenges in dementia care [9]. Non-governmental organizations based in Jordan, such as the Al Oun for Alzheimer’s Patient Care Association, have been focusing more attention on this rising public health crisis for Jordan and the MENA region [12].

Despite these trends, there is a paucity of data on dementia in Jordan and no current research specifically on dementia among Syrian refugees in Jordan. This lack of research is alarming given that many Syrian refugees might experience poor cognitive aging at disproportionate rates due to increased risk factors and growing literature highlighting potential links to increased risk of dementia [13]. One population study demonstrated an increased risk of dementia among migrants compared to native Europeans, suggesting a link between migration history and cognitive aging [14]. There is strong evidence of the relationship between post-traumatic stress disorder [15, 16] and depression [17] with dementia, both of which are experienced at high rates among Syrian refugees [18–20]. Additionally, Syrian refugees in Jordan have high rates of obesity, hypertension, diabetes mellitus, smoking, and heart disease [8, 21] which have been associated with dementia [22, 23].

Syrian refugees also face many barriers to Jordanian healthcare access including financial burden, long waiting hours, and perceived discrimination from healthcare staff [8, 21, 24]. Syrian refugees in Jordan rely on limited public, charity, and non-governmental organization facilities for their chronic condition care, which costs them around 18% of their monthly income [8]. It is difficult for Syrian refugees to maintain continuity of care and treatment within the Jordanian healthcare system [25, 26]. Additionally, given Jordan is socio-culturally very similar to Syria, with shared language, religion, traditions, and values, there are shared stigmas surrounding mental health issues and dementia, which might present an additional care-seeking barrier [27, 28]. Although the modality of care for dementia is dependent on the severity of the illness, due to religious and cultural obligations shared in MENA regions, the responsibility of dementia care largely falls to family members who lack adequate training and knowledge to care for a person with dementia [28]. Moreover, cases of cognitive impairment and dementia are often undiagnosed among minority migrants due to barriers in identifying, assessing, and managing dementia in this population [29]. To support the health of aging Syrian refugees in Jordan, research focusing on dementia knowledge and attitudes is needed to understand the unique challenges faced as a result of displacement. This study aims to document aging Syrian refugees' knowledge and attitudes toward dementia and cognitive aging, along with their experiences with the Jordanian healthcare system. Learnings from this study will provide the basis for future dementia research among this population. This study will also inform Jordanian – and other LMICs – policies and interventions to support its growing refugee population.

## Methods

### Design

This exploratory study employed a qualitative investigation into the knowledge and attitudes toward dementia and cognitive aging along with factors that facilitate or prevent access to healthcare among Syrian refugees resettled in Amman, the capital of Jordan. This location was chosen because Jordan hosts an estimated 1.36 million Syrian refugees, 90% of whom resettled in urban areas including Amman [6]. Thus, Amman is a research site that provides a strong representation of Syrian refugees resettled in an Arab country. To analyze data collected during this study, applied thematic analysis was used to capture participants’ voices as comprehensively and accurately as possible [30]. This study followed the Standards for Reporting Qualitative Research [31].

### Participants

Participants were aging Syrian refugees resettled in Amman, Jordan. Inclusion criteria were: 1) identifying Syria as a country of origin, 2) having a present or former refugee status, 3) being 55 years or older, and 4) residing in Amman, Jordan. Exclusion criteria were: 1) known diagnosis of major neurocognitive disorder/dementia, 2) inability to provide informed consent, and 3) inability to participate in the hour-long focus group.

Recruitment occurred during July 2022 through a convenience sample from Al Rafeed Center, an ethnic-based community organization partner in Amman, Jordan. Our partners advertised the study through word-of-mouth to their clients. We offered an incentive of 10 Jordanian Dinars to participate in the focus groups. Interested participants were screened, consented, and assigned to four gender-concordant focus groups. A total of 32 participants completed this study.

### Data collection

Data was collected through hour-long focus groups in Arabic by author LB, a native Arabic-speaking investigator experienced in conducting focus groups and trauma-informed interviewing. Four focus groups were held, and there were two each for the female and male groups. Focus group size ranged from six to nine participants. Demographics were collected before the start of the focus group through a questionnaire. The focus group guide (see Additional file 1) was developed by vetted field experts and direct providers after conducting a scoping review of the literature. Questions from the guide were administered in the Syrian dialect and emphasized exploring contextual factors of resettling in an Arab country such as shared culture and language. Focus groups were conducted at volunteer participants’ homes in designated rooms to maintain privacy. Focus groups were audio-recorded, transcribed in Arabic, translated into English, and reviewed for content and accuracy. All data is stored in a password-protected file hosting service.

### Data analysis

Data was analyzed through inductive thematic analysis. Investigator triangulation was used to enhance the analysis credibility. A group of investigators with access to all raw data coded the transcripts using ATLAS.ti software. To ensure dependability and confirmability, investigators met regularly to establish a codebook and intercoder agreements through the subjective assessment method [30]. Analysis was completed through meetings with coauthors both involved and not involved in coding of the data who identified recurrent themes following Crabtree and Miller’s 5-step interpretive process, and disagreements were resolved through group discussions guided by authors LB and DAK [32]. Transferability of the data is ensured by clearly outlining this study’s methodology, participant sample, and focus group guide.

### Ethical approval

The Institutional Review Board (IRB) at the University of California, San Diego approved this research (#201634). All participants provided their written informed consent to take part in this study. Local approval for data collection was approved through the community-based organization whose board is 30%, Syrian refugees.

## Theoretical framework

The socioecological model is a tiered framework focusing on the interactions between individuals, their immediate environment, and their larger social contexts [33]. This framework is widely applied in public health research to investigate how individual-, interpersonal-, community-, and policy-level factors impact attitudes toward disease and care-seeking behaviors [34]. It has also been a useful framework for previous studies on knowledge and attitudes toward dementia [35, 36]. Additionally, the socioecological model informs health promotion approaches by demanding that strategies address the individual while creating environmental conditions that promote and support healthy behaviors [37]. Thus, this model was identified as a productive framework to conceptualize the findings from this study and inform future health promotion efforts aimed at improving the cognitive health of aging Syrian refugees in Jordan.

## Results

**Fig. 1:**
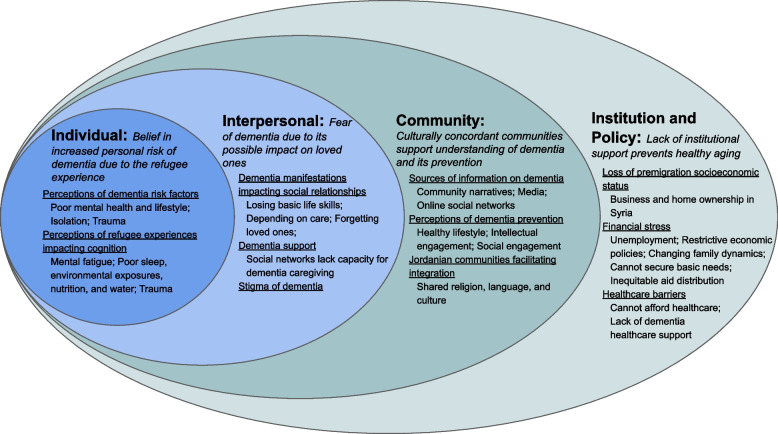
Attitudes of Syrian refugees toward dementia and healthy aging in Jordan

Demographic data of the participants (*n* = 32) is provided in Table 1. The interviews from this study yielded four themes that influence the attitudes of Syrian refugees in Jordan toward dementia and healthy cognitive aging. The themes were organized into a socioecological model of health framework (Fig. 1): 1) Individual level: belief in increased personal risk of dementia due to refugee experience; 2) Interpersonal level: fear of dementia due to its possible impact on loved ones; 3) Community level: culturally concordant communities support understanding of dementia and its prevention; 4) Institution and policy level: lack of institutional support prevents healthy aging.

**Table 1:** Participant demographics

**Characteristics**	*n* = 32	%^a^
**Gender**		
Female	17	53.1
Male	15	46.9
**Age**		
55–59	20	62.5
60–64	5	15.6
65–69	4	12.5
70 +	1	3.1
**Years since Resettlement**		
4–8	5	15.6
9	20	62.5
10	6	18.8
**Education Level**		
Illiterate	6	18.8
Less than high school	20	62.5
High school	5	15.6
Undergraduate	1	3.1
**Employment Status**		
Not Employed	31	96.9
Employed	1	3.1
**Marital Status**		
Married	28	87.5
Widowed	3	9.4
Separated	1	3.1
**Household Size**		
2–3	9	28.1
4–5	12	37.5
6–7	8	25.0
**Number of Children**		
0	1	3.1
3	3	9.4
4	8	25.0
5 +	20	62.5
**Income**		
< 100 JDs/month	17	53.1
100–200 JDs/month	10	31.3
> 200 JDs/month	5	15.6
**Economic Status**		
Not enough money to make ends meet	32	100.0
**Past Medical History**		
Arthritis	23	71.9
Hypertension	20	62.5
Diabetes	14	43.8
Other^b^	7	21.9
**Living with Dementia Relative**		
Yes	3	9.4
No	29	90.6
**Family History of Dementia**		
Yes	3	9.4
No	29	90.6
**Family Members with Dementia**		
Mother	1	3.1
Father	1	3.1
Both	1	3.1
NA	29	90.6
**Self-Rated Overall Health**		
Poor	6	18.8
Not good	10	31.3
Average	16	50.0
**Self-Rated Memory**		
Poor	2	6.3
Not good	9	28.1
Average	10	31.3
Good	7	21.9
Excellent	4	12.5

^a^Percentage may be less or greater than 100.0 due to participants electing not to answer questions and selecting multiple choices, respectively

^b^Includes: osteoporosis, eye pain, cardiac disease, back pain, asthma, benign prostatic hyperplasia, and not applicable

### Individual level

Participants described a range of variables they believed to be risk factors for developing dementia and overall poor cognitive aging. These factors include genetic predispositions, environmental factors such as lack of clean water and air, cardiovascular disease, diabetes, and nerve damage. They also reported poor lifestyle as an important risk factor for developing dementia and placed a particular emphasis on poor environmental exposures:

*“If we eat wrong foods, we don’t move, we’re not exercising, we’re not walking, we’re not learning stuff, we’re smoking or inhaling secondhand smoke... this opens up [the possibility of developing* *dementia].”*

Age was a contested risk factor for dementia. Some participants believed that dementia was a risk to all aging people while others felt that dementia was neither a normal process of aging nor a risk factor for it:

*“I think about my dad because he had Alzheimer’s disease at age of 50. So no, [dementia] has nothing to do with aging.”*

All participants, however, shared a strong belief that poor mental health, trauma, and isolation are important risk factors for developing dementia. Many cited how loss from war, anxiety, depression, and loneliness are detrimental to cognitive health:

*“Trauma, wars, pain, and depression. Fear is a major risk [for dementia].”*

*“The place we live in also impacts [dementia risk]. All the things we’ve been going through, all the war, it’s influencing us physically and mentally.”*

As a result of these risk factors, they believed they are more likely to develop dementia and many reported concerns of poor cognitive functioning. This increased personal risk stemmed specifically from their refugee experience:

*“We have fear! Fear from what we heard and saw. Rape. Killing and slaughtering kids. Infants! This is not easy. Imagine the mother who witnessed these events. How can she continue life after experiencing all of that? Every household in Syria is hurt from the inside. Because of those experiences people are getting so much more besides mental health issues.”*

*“You sleep with horror.”*

They believed that their experiences with painful memories and consistent worry lead to cognitive damage:

*“They say overthinking is what makes someone’s brain explode. Like the phone, if you store a lot of stuff in it, you would have to delete other information to make space. Our brains are the same.”*

Participants also placed an emphasis on their increased risk of dementia due to poor nutrition and lack of access to clean water:

*Because we are experiencing more stress and malnutrition, of course [we have a higher risk of dementia]. Everything is deficient when we did some tests… vitamin D, B12… all of it.”*

Overall, although none of the participants were diagnosed with dementia, they endorsed cognitive decline such as worse memory. They often compared themselves to the older relatives they used to know and felt that, even at a significantly younger age, their cognitive performance is weaker. They related this early aging to their refugee experience:

*“The worry made us age. Here, we feel one year equates to four of five years from when we were living in our home country.”*

### Interpersonal level

As aging refugees, participants were afraid of developing dementia, particularly those endorsing cognitive decline, early aging, and/or a family history of dementia. Participants feared losing their knowledge, basic life skills, and independence. These possible changes troubled participants because of the potential impact on their loved ones (i.e., needing to depend on their family for care, and no longer being able to recognize or remember loved ones):

*“You are scared because you wouldn’t know your children or wouldn’t realize to take your medication. You wouldn't know anything anymore. You would see your relative in the street and not know him.”*

Although participants believed that their social support networks could learn caretaking skills for them if they ever developed dementia, they also acknowledged that their social networks would not have the capacity to support them. They attributed this barrier to dementia support to mainly socioeconomic stress:

*“We are still supporting people back in Syria. Everyone has their own problems. We cannot help [other Syrian refugees in Jordan] and they can’t help us.”*

Finally, some participants described the stigmatization of dementia and feared burdening their loved ones with stigma and social shame. There was a fear of being labeled “crazy” and outcasted by community members. Participants were significantly impacted by such a label:

*“Here [Syria or Jordan], we perceive having dementia as being crazy or insane. Everyone who is diagnosed with that is considered crazy. We don’t think that having dementia or Alzheimer’s is totally fine. Instead, we avoid that person.”*

*“In Syria, my shoulder and ankles would hurt so my doctor gave me psychiatric medication and anesthesia medicine. People there told me that this medication was for crazy people. I got very sad.”*

### Community level

Participants described their local communities and online communities as important sources of information on dementia and healthy aging. Their understanding of dementia was shaped by social media, narratives from community members and family members, school, news, generational wisdom, and healthcare providers:

*“I know good sleep is also good [for cognitive health]. People in the old days would tell you to sleep early.”*

*“I went to school for biology and that’s where I heard about Alzheimer’s before. Of course, people who haven’t gone to school would learn about dementia from their friends and their family... the environment and the community around a person.”*

Participants’ understanding of dementia, particularly perceptions of dementia symptoms, was largely shaped by the experiences of those in their community with dementia. Participants learned to associate specific signs and symptoms with dementia such as memory loss, losing knowledge, eccentric behaviors, and declining ability resulting in a child-like dependency:

*“Someone starts to forget, not knowing anything, losing their brain. I had my cousin in Syria who used to put a blanket on her shoulder saying I’m going to bring my son. She would get mad, take things like shoes and put them somewhere else. She wouldn't know anything and she would go to random places. My dad was like that too.”*

*“This is the thing that frightens someone the most. You feel like a person [with dementia] becomes a child again.”*

Participants were also asked about their understanding of dementia prevention and what factors decrease their risk of developing dementia. Many reported that a healthy lifestyle – including nutritious diet, physical activity, intellectual and spiritual engagement, and good sleep hygiene – can prevent the development of dementia:

*“My husband reads the Quran twice a week. If it was not for it, he could have forgotten everything.”*

Additionally, good mental health and feeling connected to others were cited to prevent dementia. Participants mentioned how resettling in an Arab country with shared cultural ties and religion has supported their mental health and therefore decreases their risk of developing dementia:

*“Here [Jordan] it was easy to integrate into the society because of the similar culture and language. Here we do not feel alienated. Everything is the same, like traditions and foods.”*

*“[When we hear the call to prayer], we rest and feel better. An Islamic country is just different.”*

Despite knowing that resettlement in Western countries might provide better socioeconomic security, some participants continued to prefer an Arab country for resettlement because of fear of acculturation, losing communities, language barriers leading to isolation, and losing religious ties. They described that the contextual factors of living in Jordan that help in preventing dementia would be lost if they migrated to a culturally discordant community:

*“My son in Europe tells me if he has a chance to come here [to Jordan] he will do so because it’s better for him. Here, people understand each other through the language. In Europe, he doesn’t feel comfortable,”*

### Institution and policy level

All participants reported poor socioeconomic conditions after resettlement which have caused significant stress and burden. They stated that premigration socioeconomic status and health were significantly better than their current situation. They attributed this financial stress and burden to their loss of home and business ownership in Syria in addition to restrictive economic policies that prevent employment:

*“75% of your income goes to your rent and electricity. In Syria we owned our houses. Over there, we did not have to rush to pay rent.”*

*“Us Syrians, we like to work. Everywhere we go, we have plans and we open businesses. Here in Jordan, we can’t do anything without talking to people and getting things approved. We are not allowed to buy cars or houses or get a store.”*

*“I was not able to work in healthcare because they do not allow it here in Jordan. They prefer to employ Jordanians than people from other countries.”*

Participants complained of rent burden, unemployment, and rising costs that led to an inability to secure basic needs. They were also concerned about the changing family dynamics as a result of poor socioeconomic conditions, forcing children to be the main income source rather than parents. This constant source of worry and an inability to engage in healthy lifestyles, such as consuming nutritious food, prevented healthy aging:

*“We always fear emergency. You are running around all day and some people do not even have anything to eat.”*

*“A lot of people are trying to get their kids married but they are not able to because they don’t have money. They cannot get a house for their kids. The parents are the ones depending on their kids and a lot of problems are happening like divorces. Those are the things we’re suffering from, and all of this can cause dementia.”*

While most participants described receiving financial aid, all mentioned that the aid did not suffice. The aid, called “coupons,” was not able to support participants to meet their basic needs:

*“People in immigration are not helping you with money and food. You are sitting here in Jordan and the government can barely provide for its own people. We cannot go back to Syria because we are scared. Here, we are mostly safe, but we don’t have money and we are tired. That’s hard.”*

Moreover, some participants mentioned that their aid was either stopped or reduced when aid organizations deemed they no longer qualified for it. Participants complained of this sudden loss of financial support and its detriment to their lives. They attributed the lack of financial support to flaws in the administrative system and corruption of the organizations:

*“My doctor told me I have too much fat in my blood. He told me I could get a heart attack any minute. How can I control this? I don’t have healthy food to eat because they haven’t given me food coupons for a year now. They said your family does not qualify. Who qualifies then? What is a qualifying family?”*

*“[Representatives from aid organizations] come to your house and they ask you if you have a refrigerator. ‘How much do you eat? Do you eat meat?' And then they give you the coupons based on that. They come to your house and see what you have missing and they* *try to provide you with that. But if you happen to have a fan in your house that you got from your friend as a gift, they’ll see that and deem you unqualified and stop giving you aid. Over a fan.”*

*“The aid organizations won’t give coupons to a struggling family like us, but you’ll see a family with a nice car receiving coupons. If the system was fair, how would things like this continue to happen?”*

This lack of institutional support also manifests as significant barriers to healthcare including lengthy commutes and discrimination from staff. Participants talked about being unable to afford healthcare visits and life-saving treatments. A group of participants in one focus group described how the inaccessible medications have forced them to develop a medicine exchange program to sustain their necessary treatments:

*“For example, I take my medications but there's one type I do not want to take. So, I would try changing with another person. So, we create a group of 10-15 people to gather* *and exchange medications. If someone has extra medication, he provides it to whoever is in need. For example, [one] person here does blood work every 6 months. It turns out that he does not need the diabetes medication anymore... [So he would give it to whoever is in need].”*

Finally, participants reported that the Jordanian healthcare system has failed to meet their health needs as an aging population, including their mental and cognitive health:

*“I don’t think [I will be supported if I ever get dementia]. That is impossible. They are not even supporting our physical health. They are not even supporting our mental health.”*

## Discussion

This study examined the knowledge and attitudes of Syrian refugees resettled in Jordan on dementia and cognitive aging through a socioecological model. To the best of our knowledge, this is the first study to focus on dementia among Syrian refugees. Findings highlight that participants’ beliefs and attitudes toward dementia are impacted by complex interactions of personal, interpersonal, community, and larger institutional/policy factors. Overall, participants’ understanding of dementia risks and their self-awareness in recognizing personal risk factors for poor cognitive aging are concordant with the literature. Similarly, participants’ awareness of documented protective factors underscores the benefits of resettling in a neighboring Arab country that facilitates community integration. However, lack of institutional support and beneficial refugee policies remain a barrier to healthy cognitive aging and overall wellbeing for this population. These themes are echoed in previous qualitative research on attitudes toward dementia and cognitive aging among migrants and refugees [38–40].

Current dementia literature supports the risk factors cited by participants. Multiple studies confirm their belief in poor mental health as dementia risks [15, 16, 41, 42]. Further risk factors endorsed by participants were supported by the latest Lancet Commission Report on Dementia Prevention, Intervention, and Care including social isolation, obesity, physical inactivity, and air pollution [43]. Therefore, participants are correct in identifying themselves to be at an increased risk of dementia when endorsing their poor mental health, lifestyle, and environment, in addition to their demographic risk factors (e.g., low education, hypertension, diabetes) (Table 1) [43]. Moreover, 34.4% of participants rated their memory as poor or not good (Table 1), and studies demonstrate cognitive deficits in older adults with subjective memory complaints [44].

Although Jordan currently lacks a separate National Dementia Plan recommended by the Alzheimer’s Diseases International [45], it is among the few LMICs that incorporated dementia into existing national plans. When Jordan launched its first National Mental Health Policy in 2011 and National Mental Health and Substance Use Action Plan in 2018–2021, it declared dementia as a priority condition [46, 47]. In both these plans, Jordan has emphasized the importance of guaranteeing refugees “free access” to mental health support and interventions [46, 47]. While it is promising that Jordan is actively supporting refugees’ mental and cognitive needs, more effort is needed to thoughtfully incorporate the refugee experience into Jordan’s future nationwide health plans. As Jordan develops its National Dementia Plan, five key recommendations were identified from the study results to support Syrian refugees’ cognitive aging (Table 2). Since the socioecological model was applied to this study, these recommendations were developed to promote favorable changes at the individual level while concurrently creating favorable resettlement conditions for aging Syrian refugees.

**Table 2:** Recommendations for the Jordanian Government to include in its National Dementia Plan to address the cognitive needs of aging Syrian refugees

Recommendations	Objectives	Action Items
Advance research to understand the cognitive needs of aging Syrian refugees	Advancing scientific research and international collaboration to improve the understanding of dementia prevalence, risk factors, protective factors, and effective interventions among Syrian refugees will build the foundation for preventing dementia within this population	- Conduct dementia epidemiological studies in the Syrian refugee population - Develop and implement tailored interventions to prevent cognitive decline - Ensure parallel studies for refugees in camps and/or inclusion of encamped refugees in future research
Reinforce awareness of the risk and protective factors of dementia	By reinforcing the correct knowledge Syrian refugees already have on dementia risk and protection, they will be empowered to take action to protect their own health and reduce their risk of developing dementia	- Leverage existing sources of health information and generational wisdom (older community leaders as cultural assets) - Implement dementia stigma reduction strategies - Develop feasible recommendations for dementia protection
Invest in healthy/age-friendly housing and neighborhoods	Syrian refugees, like any other group, must be supported by housing options and environments that foster healthy lifestyles, allowing them to engage in healthy behaviors	- Support community garden projects - Implement smoking cessation programs - Provide community mental health and psychosocial support - Encourage social interaction through spiritual spaces - Encourage walking and other forms of physical exercise - Offer inspection and housing options for household crowding
Remove healthcare barriers to promote care-seeking behaviors	Improving access to care can decrease chronic diseases that are risk factors for dementia, and incentivize Syrian refugees to discuss their cognitive aging needs and dementia prevention with their healthcare providers	- Subsidize cost of healthcare utilization - Protocolize dementia screenings during doctor visits, especially in primary care - Expand mental health services - Train healthcare staff on anti-discrimination strategies
Expand assistance and funding to support basic living needs	By allowing Syrian refugees to meet their basic needs, they will develop the capacity to engage in healthier lifestyles and build cognitive reserve to support healthy aging	- Provide unconditional cash transfers - Invest in affordable housing - Establish and allow for employment opportunities (without the need for work permits/sponsors)

### Advance research to understand the cognitive needs of aging Syrian refugees

Dementia research among refugee populations remains extremely limited. Gaps include epidemiological studies quantifying prevalence and incidence of dementia among Syrian refugees in Jordan along with pertinent risk factors that may be unique to this population. Rigorous studies on dementia prevention are also important. Such studies could replicate previous randomized controlled trials that demonstrated the efficacy of lifestyle interventions on cognitive decline prevention, such as the Finnish Geriatric Intervention Study to Prevent Cognitive Impairment and Disability (FINGER) [48]. The FINGER model has already been adapted and tested in different countries within the newly launched World Wide FINGERS network [48]. By joining this network, Jordan could lead the research effort on dementia prevention among LMICs in the Middle East and be the first to focus on dementia prevention among the refugee population. Future research should also focus on family and caregivers of Syrian refugees with dementia, especially since previous research in MENA communities has shown family caregivers are ill-equipped to support their patients [28].

There are research funds available from Jordan's Ministry of Higher Education and Scientific Research to support such endeavors, especially since declared national research priorities clearly align with healthy refugee cognitive aging [49]. Importantly, research efforts on cognitive aging among Syrian refugees should incorporate encamped Syrian refugee populations. Although they only represent around 10% of the Syrian refugee population [6], this subset has unique health needs and exposures [50], and therefore efforts are needed to ensure their representation in national dementia research.

### Reinforce awareness of the risk and protective factors of dementia

The Lancet Commission Report on Dementia underscored the importance of identifying potential modifiable risk factors for dementia to inform public health efforts to prevent cognitive decline [43]. Dementia literacy is necessary for individuals to engage in dementia prevention behaviors, and the World Health Organization has created an initiative to improve dementia literacy [51]. While participants had a good understanding of risk and prevention factors for dementia, it remains important to reinforce and promote this accurate awareness. Initiatives should leverage the existing sources of information within the Syrian refugee community including social media networks and local community organizations focused on dementia [12]. For example, utilizing faith networks for dementia education and cognitive screening can improve knowledge and promote preventative care seeking [52]. Since participants cited dementia stigma as an area for improvement, educating the community on dementia facts can reduce dementia stigma and improve quality of life for people with dementia [53].

### Invest in healthy/age-friendly housing and neighborhoods

Neighborhood-built environments are pertinent to older adults’ cognition, memory, and dementia [54], thus investing in healthy and age-friendly neighborhoods is essential for preserving cognitive reserve among Syrian refugees and the health of those already impacted by dementia. Green space exposure is the most well-studied domain of the built environment and multiple studies demonstrate its protection against cognitive impairment and dementia [54]. Creating community garden spaces for Syrian refugees can have multiple benefits that protect from dementia including improved fruit and vegetable intake, reduction in perceived stress and loneliness, and overall improvements in quality of life [55–58]. This is important given that many participants attributed their deteriorating health post-resettlement to poor nutrition due to food insecurity. Many shared their experiences growing produce in their home gardens in Syria, and that source of nutritious foods has been lost for them in Jordan. Therefore, investing in community gardens for the Syrian refugee community is a promising health promotion strategy with multidomain lifestyle benefits.

Other community projects include smoking cessation programs to reduce exposure to second-hand smoke and addressing air pollution as a dementia risk factor [43, 48]. Public health efforts should increase awareness and accessibility of Jordan’s preexisting smoking cessation services [59] and tailor those programs to the needs of Syrian refugees [60]. Further interventions include promoting community mental health services and training community leaders and peers to support mental healthcare access [61, 62]. Since many participants endorsed spirituality as a source for intellectual engagement and mental health support, investing in faith-based community programs for dementia prevention is worthwhile. Faith-based community interventions can improve mental health parameters and reduce feelings of isolation among older adults [63, 64]. Isolation can also be improved by supporting housing spaces that are conducive to multigenerational living, which is associated with reduced depressive symptoms among older adults [65].

While developing projects to improve the communities housing refugees, codesign of these initiatives is fundamental. It is clear from this study’s results that participants are interested in improving their living conditions to take care of their health and the health of their loved ones. Offering them opportunities to partner with healthcare providers and public health experts to codesign community improvement projects can enhance their sense of agency and connectedness to the Jordanian community.

### Remove barriers to healthcare to promote care-seeking behaviors

Participants faced many barriers to healthcare, preventing them from treating their chronic conditions and seeking care for their cognitive health. While Jordan has focused on strengthening its health sector, Syrian refugees resettled in Jordan continue to face barriers to their healthcare access [8, 21, 24]. Since March 2019, Syrian refugees access public health services at a non-insured Jordanian rate (subsidized 80%) [6], yet financial burden remains a significant healthcare barrier [8, 21, 50]. All participants from this study reported they did not have enough money to make ends meet (Table 1) and many were unable to secure basic life-saving medicine such as insulin for diabetes. Therefore, continued subsidization and increase in free healthcare services are necessary for the Syrian refugee community. Dementia care is costly, and as Middle Eastern countries are expected to experience the highest increase in dementia cases [9], early detection of dementia and management of comorbidities are essential for reducing costs [66]. It is also important to ensure Syrian refugees’ utilization of mental health services. Jordan should continue to expand and provide free mental health services for Syrian refugees as it has already outlined in its national mental health plan [47]. Additionally, participants revealed experiences of discrimination within the healthcare system. This has previously been reported as a barrier to Jordanian healthcare [24], highlighting the need for anti-discrimination strategies such as staff training to reduce this strain on refugees.

### Expand assistance and funding to support basic living needs

Overall, participants felt that it was difficult to age healthily when they are unable to survive in the present. Despite 81.3% of participants resettling for 9 or more years in Jordan, severe financial stress burdens this community (Table 1). While Jordan and non-governmental organizations provide financial aid to refugees, it is evident that this aid is simply insufficient [8, 50]. Public health experts of Jordan have already called for revisiting cash assistance criteria, increasing the quality and quantity of food coupons, and increasing legal work opportunities for Syrian refugees [50]. Unconditional cash transfers are associated with positive health outcomes in humanitarian settings [67] and are warranted for the Syrian refugee community. Furthermore, many participants expressed rent burden as a major financial stressor, and therefore investment in affordable housing programs is also warranted. Securing funding for expanded assistance to Syrian refugees in countries with limited resources such as Jordan remains a challenge. The Jordanian government relies on current partnerships with humanitarian/non-governmental organizations including the United Nations High Commissioners for Refugees and the United Nations International Children’s Emergency Fund [47]. However, such forms of financial assistance are not sustainable. Interventions to address the poverty rates of refugees in Jordan (i.e., lifting restrictions for refugees to work, microfinance interventions) for the long term are urgently needed. Additionally, a life-course perspective is important when addressing the burden of aging as a refugee [68]. For example, improving education and employment opportunities during early and mid-life stages can improve socioeconomic status and relieve significant stress and cognitive decline with age [43].

## Strengths and limitations

This study has a few limitations. Participants were recruited by convenience sampling which may limit the representativeness of the study and the generalizability of the findings. Moreover, this study focused on refugees resettled in Amman and did not include the experiences of refugees in Jordanian camps or other cities in Jordan. While most Syrian refugees in Jordan are resettled in urban areas such as Amman [6], it is important to note that results from this study may not reflect the narratives of encamped Syrian refugees, and a parallel study in that population is necessary. There is a possibility of self-selection bias as individuals with strong opinions on dementia, such as those concerned about their cognitive decline, may have been more likely to participate. Finally, as dementia and poor cognitive decline may be stigmatized within this community, social desirability bias may have prevented participants from disclosing certain experiences or opinions on the topic. Nevertheless, investigators encouraged participants to share their authentic ideas and experiences to reduce this bias.

## Conclusions

Despite having knowledge of dementia risk and protective factors, Syrian refugees resettled in Amman, Jordan struggle to engage in lifestyles to support healthy cognitive aging. Poor socioeconomic status and poor mental health resulting from the refugee experience have led to challenges with food insecurity, unemployment, household crowding, healthcare access, chronic disease management, and overall wellbeing. As Jordan develops its National Dementia Plan, it must identify and implement dedicated strategies for its Syrian refugee population, leading the way for other nations to address refugees and other minoritized groups in their National Dementia Plans.

### Supplementary Information

**Additional file 1. **Focus group guide.

## Data Availability

The datasets used and/or analyzed during the current study are available from the corresponding author on reasonable request.

## Abbreviations

LMICs: Low- and middle-income countries
MENA: Middle East and North Africa
