# Supplementary material for: Attitudes toward dementia and cognitive aging among Syrian refugees resettled in Jordan: a qualitative study
Source: BMC Public Health. 2023 Nov 22;23:2307. doi: 10.1186/s12889-023-17183-5 (PMC10664261; doi:10.1186/s12889-023-17183-5)
Supplement: Supplementary file 1 — Additional file 1. Focus group guide. [file 12889_2023_17183_MOESM1_ESM.docx]

**Additional File 1:**

File format .docx for Focus Group Guide which includes the questions that guided the conversations with participants.

| **Focus Group Guide** |
| --- |
| 1. We are all here because we want to discuss Alzheimer’s disease (AD). Can you tell us what comes to mind when thinking about dementia or AD? What do you know about it?   - PROBE: What puts people at risk for getting dementia? What makes people develop AD? - PROBE: Describe how you go about getting information about AD? Family? Doctor? Media? - PROBE: Do you think there is a connection between cardiovascular disease and AD? |
| 2. Tell me, do you believe that AD can be prevented (why/why not)? If yes, how? |
| 3. What do you think one can do to reduce the risk of or prevent AD? |
| 4. What comes to mind when you hear someone you know has AD or a related dementia?   - PROBE: Describe the kind of thoughts you have when you hear someone has AD or a related dementia? Why? - PROBE: Tell me what scares/worries you about cognitive disorders? - PROBE: How does it make you feel (when participants described their experiences with people affected by cognitive disorders)? |
| 5. Thinking about your own history as a refugee, what is it about the refugee experience that can affect health?   - PROBE: What specific things about the refugee experience may affect someone’s risk of developing AD? - PROBE: What specific things about the refugee experience may affect how much you know about AD? |
| 6. Thinking about your health information sources and the type of healthcare you receive here in Jordan, tell me about the healthcare and health information you receive?   - PROBE: Tell me about the place you go to receive care. How does it compare to care in other countries where you have lived before? - PROBE: Describe how you go about getting information on healthy aging? - PROBE: How would you describe your experience with your healthcare providers? How about when you seek sources of information? - PROBE: Are you able to have continuity care with a provider? What factors facilitate or discourage this? - PROBE: How familiar are you with procedures of accessing care? (e.g., do you know which centers you can visit? Do you know what documentation you need to bring with you?) |
| 7. What healthcare services would support you if you had dementia? (e.g., doctor, nurse, community health worker, nursing facility, hospital, clinic)   - PROBE: How would this be different if you were resettled in a non-Arab country? |
| 8. What social support would care for you if you had dementia? (e.g., Family (children, siblings, cousins), friends, religious community, etc.)   - PROBE: How would this be different if you were resettled in a non-Arab country? - PROBE: How is this social support different from what you would have received prior to migration? - PROBE: Do you think your social support would have the appropriate knowledge/experience to provide you care? |
| 9. What do you believe is your likelihood of developing dementia?   - PROBE: How does being a refugee impact your likelihood of developing dementia? Do you think it will be different if you are resettled in a western or non-Arab country? - PROBE: Does sharing a language, similar culture, or religion influence your dementia risk? If yes, how so? |
| 10. What motivates you to do things to prevent getting AD? |
| 11. What stops you from changing your health behavior and lifestyles? |
| 12. What support would be most beneficial in helping you prevent dementia? |
